# Investigation of the Sensitivity of Acoustic Emission to the Differentiation Between Mode I, II, and III Fracture in Bulk Polymer Materials

**DOI:** 10.3390/polym17010125

**Published:** 2025-01-06

**Authors:** Ali Shivaie Kojouri, Dimitrios G. Aggelis, Javane Karami, Akash Sharma, Wim Van Paepegem, Danny Van Hemelrijck, Kalliopi-Artemi Kalteremidou

**Affiliations:** 1 Department of Mechanics of Materials and Constructions (MeMC), Vrije Universiteit Brussel, B-1050 Brussels, Belgiumdanny.van.hemelrijck@vub.be (D.V.H.); kalliopi-artemi.kalteremidou@vub.be (K.-A.K.); 2 Department of Materials, Textiles and Chemical Engineering, Ghent University, Technologiepark–Zwijnaarde 46, B-9052 Zwijnaarde, Belgium

**Keywords:** acoustic emission, fracture, crack, mode I, mode II, mode III, polymer fracture

## Abstract

There is very limited research in the literature investigating the way acoustic emission signals change when polymer materials are undergoing different fracture modes. This study investigates the capability of acoustic emission to recognize the fracture mode through acoustic emission parameter analysis, and can be considered the first-ever study which examines the impact of different loading conditions, i.e., fracture mode I, mode II, and mode III, on the acoustic emission parameters in polymer materials. To accomplish this, prism-like pre-cracked polymer specimens were tested under the three different fracture modes. Acoustic emission parameters appeared sensitive to the different loading conditions of the pre-cracked specimens, indicating that acoustic emission can be used to distinguish the three fracture modes in polymer materials. Both frequency and time parameters reflect changes in the stress states at the crack tip. The duration and rise time of the waveforms were found to be the most sensitive acoustic emission parameters for identifying the fracture mode, while the average frequency variation can be employed to differentiate between in-plane and out-of-plane fracture modes. In order to interpret the experimental results in relation to wave mechanics, numerical wave propagation simulations for longitudinal and shear excitations were performed to simulate tensile and shear fracture modes and the corresponding emitted waves. An interesting correlation between the experimental and numerical results exists, showcasing acoustic emission’s potential for fracture identification.

## 1. Introduction

Non-destructive testing is used by engineers for inspecting structures prior to the final failure. There are different non-destructive testing methods based on ultrasonics, acoustic emission (AE), magnetic flux leakage, eddy currents, and radiographic methods [1]. AE is regarded as a distinctive non-destructive approach since it allows for the monitoring of degradation throughout the loading phases of structures and due to its sensitivity to cracks and dislocations at the early stages of the loading [2,3]. This interesting characteristic of AE makes it a powerful tool for studying the fracture phenomena and damage mechanisms in different materials and geometries.

AE has been employed by researchers to investigate the damage characterization of the composite materials [4,5,6,7]. Sobhani et al. [8] examined the buckling behavior of E-glass composite using AE. They classified the damage mechanisms of the composite during buckling using the Gaussian mixture model. Mohammadi et al. [9] predicted the crack growth of double cantilever beam specimens under mode I loading conditions by fitting a linear equation to the cumulative energy of AE. Guo et al. [10] used AE to determine the crack initiation and the corresponding load for the evaluation of the fracture toughness of a wood-plastic composite. Heidary et al. [11] and Mohan et al. [12] used the gradient of cumulative counts and the gradient of cumulative energy plots to detect the damage of different composite specimens under tensile loads. Fotouhi et al. [13] tested different composite-made pre-cracked specimens under mode I, mode II, and mixed mode I–II in order to characterize the damage mechanisms in composite materials using AE. Their AE results were in good agreement with Scanning Electron Microscope (SEM) observations. Besides static loading, AE can be also employed for damage assessment of composites under dynamic loading, i.e., fatigue loading and impact [14,15,16]. Kalteremidou et al. [15] used AE in carbon composite hollow beams under fatigue loading to characterize the damage mechanisms. They detected damage that was not detectible by visual inspection. Additionally, by analyzing the rise time and average frequency they differentiated the damage modes in the tested specimens. AE has also been utilized to monitor adhesive joints [17,18,19,20]. Dzenis et al. [21] first classified AE activities in fracture tests conducted under pure mode I and mode II loading conditions. Then they compared the AE data from mixed mode single-lap joints to the pure mode I and II cases and concluded that the activity of the single-lap joints was resembling more the mode II AE features. Saeedifar et al. [22] demonstrated that AE is capable of detecting damage in adhesive joints at the early stages of loading. Furthermore, by classification of damage from AE activities, they showed the contribution of different damage mechanisms to the final failure of the joint. Du et al. [23] employed a wavelet transform-based method for localization and identification of the matrix cracking and delamination in carbon fiber-reinforced plastic composites.

Apart from composite materials, AE is used to study damage mechanisms and self-healing processes of concrete, cementitious composites, stones, and masonry structures [24,25]. Liu et al. [26] conducted experiments on single-edge notched bending specimens made from granite and analyzed the AE parameters. Their study revealed that the tensile mode was dominating the fracture process of granite. Additionally, the portion of tensile failure appeared to rise by increasing the specimen size. Livitsanos et al. [27] monitored fracture of white clay bricks by AE under either shear or tensile-dominated conditions. They showed that tensile and shear fractures had different AE signal characteristics and that the fracture mode can be accurately assessed in laboratory conditions. Farhidzadeh et al. [28] classified AE parameters of cementitious materials using support vector machine which is known as a supervised pattern recognition algorithm. The lowest error in their classification was achieved by using average frequency and rise angle. They also discussed the effect of propagation and sensor distances on the classification error.

Polymer composites are used in different applications, from wind turbine blades to aerospace parts. The polymer’s properties can significantly affect the subsequent fracture response of the composite. Polymer material damage [29,30], plasticity [31], and fracture [32,33] have been investigated using AE by many researchers. Casiez et al. [31] studied the damage and plastic deformation of polyethylene under tensile loads using AE. By analysis of the AE signals during the tensile test, they could detect the initiation of the plasticity and/or damage. Furthermore, they concluded that necking causes diffusion of the acoustic energy and hinders the detection of AE signals. Moreover, brittle and ductile polymers showed different fracture behaviors under loading. In order to study the fracture behavior of different polymers used for dental application, Skal’s’kii et al. [33] employed the AE technique and signals were analyzed using wavelet transform. They detected the ductile, brittle, and ductile–brittle behavior of different polymers at several loading stages from AE signals. Heinzmann et al. [32] used AE to investigate the fracture source mechanism in pre-cracked PMMA under mode I loading conditions.

Besides experimental studies, wave propagation simulations are used as a powerful tool to understand the physics behind it and help to interpret the AE results [34,35,36]. Guo et al. [34] used numerical simulations to investigate the effects of voids and sensor positions for defect detection. They showed that frequency and time domain parameters were sensitive to the existence of voids in case of using perfect sensors, which were defined at a single node and were sensitive to all frequencies. However, the presence of voids did not have a major impact on the data received by the R15 sensors, which are narrow-band resonant sensors with a high sensitivity, commonly used in AE. The sensors were modelled as actual ones, including aperture and surface contact effects as well as frequency sensitivity, pinpointing the complexity and difficulty of the AE parameters analysis. In order to train the T-delta mapping algorithm to identify the AE source location in a steel plate with four circular holes of different radius at different positions, Yang et al. [36] simulated the plate using the finite element method, which decreased significantly the time and labor needed to collect and process the AE data. To better understand the influence of the damage source in the AE parameters, Hamam et al. [35] simulated fiber breakage and fiber-matrix debonding using also the finite element method.

**Figure 1:**
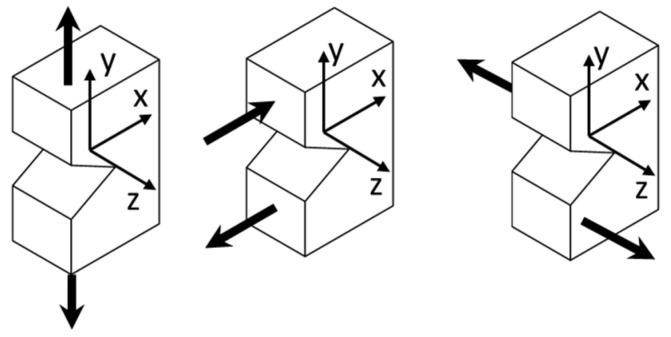
Schematic of different fracture modes acting at the crack front, from left to right: mode I or in-plane opening, mode II or in-plane shear, and mode III or out-of-plane shear.

There is a limited number of setups and fixtures to perform fracture tests under the three pure modes (mode I, II, and III as shown in Figure 1) and mixed-mode fracture using the same specimen geometry [37,38]. Asymmetric four-point bending has been used successfully to perform fracture tests under pure mode II [39] and mode III [40,41]. One advantage of asymmetric four-point bending for conducting fracture tests under modes II and III is the applicability of simple specimens, i.e., pre-cracked prism-shaped specimens. Using the same geometry, the mode I fracture test for polymer materials can be conducted utilizing three-point bending [42].

Improvement of existing structures requires the detection and identification of various failure mechanisms. There are different experimental tools used by engineers to monitor structures under different loading conditions such as Digital Image Correlation (DIC), strain gauges, and AE. However, for employing such tools in real applications, their capabilities have to be first examined under controlled conditions, like laboratory tests. The studies mentioned in the previous paragraphs show the versatility of acoustic emission for damage assessment and monitoring of specimens and structures under different loading conditions as well as for different materials. However, to the best of the authors’ knowledge, there are no attempts in the literature to study the capability of AE in distinguishing the fracture mode of polymer materials under in-plane and out-of-plane loading conditions, i.e., mode I, mode II, and mode III. To do so, an epoxy-based adhesive developed for the wind turbine industry is used in this study for sample preparation and testing. Indication of the dominant fracture mode under certain loading conditions of wind turbine blades might be beneficial during the development stages of wind turbines towards their optimization and the establishment of more reliable numerical simulations. Therefore, in the present study, the capability of AE is examined for detecting the fracture mode of pre-cracked polymer samples, tested under different fracture modes and using the same sensor positions and sample geometry to facilitate the interpretation of the results. Additionally, numerical simulations are used to interpret the results of the experiments. Specimens’ preparation, experimental setup details, a brief introduction to acoustic emission, and discussion of the results are provided hereunder.

## 2. Materials and Methods

### 2.1. Material and Specimen Preparation

**Figure 2:**
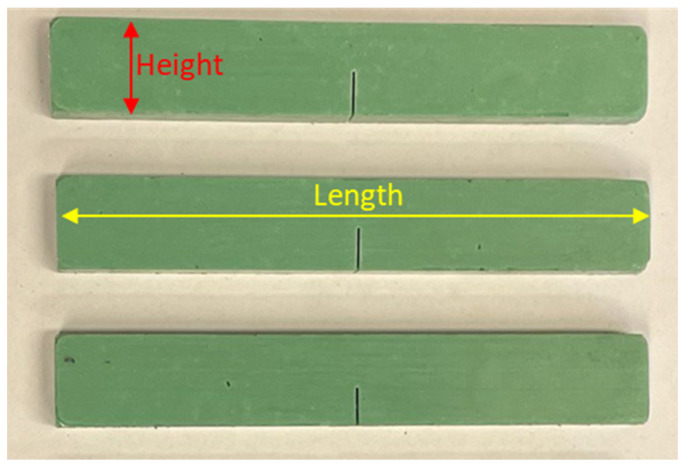
Pre-cracked prism-shaped specimens manufactured using Sika power^®^-830 (Sika Technology AG, Zürich, Switzerland) for the fracture tests.

The Sika power^®^-830 (Sika Technology AG, Zürich, Switzerland), an epoxy-based adhesive specifically designed for the wind turbine blade industry, was utilized in this study for manufacturing fracture test samples. It is a two-component adhesive with a mixing ratio of 47:100 by weight. Resin and hardener were mixed in the recommended weight ratio using a mechanical stirrer for specimen manufacturing. After 10 min of mixing, the mixture was put under vacuum for 10 min to degas it. The demolding agent Sika liquid wax-815 (Sika Technology AG, Zürich, Switzerland) was utilized for the production of bulk adhesive samples. After transferring the material to the mold, it was cured at 70 degrees Celsius for four hours. The mold was left in the oven to cool down to prevent specimens from experiencing a thermal shock. The final specimen had dimensions of 130 mm length, 20 mm height, and 10 mm thickness. Afterwards, an 8 mm long pre-crack was machined in each sample as shown in Figure 2, depicting the pre-cracked specimens made of pure adhesive. The pre-crack was then sharpened by a fresh razor blade to a length of 2 mm; hence, the ultimate crack length was equal to 10 mm. The mechanical properties of the adhesive, which were determined from tensile tests [43], are enumerated in Table 1.

### 2.2. Experimental Setups

**Figure 3:**
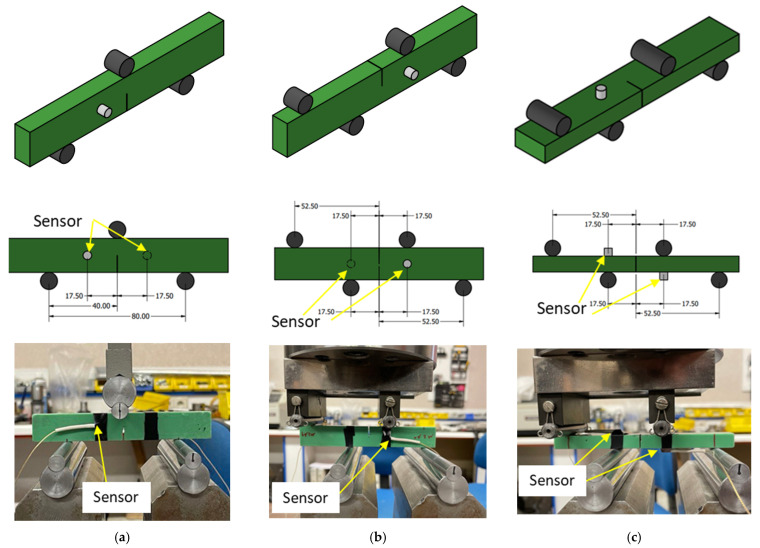
Schematic of specimens, location of the supports and sensors, and experimental setups for (**a**) mode I, (**b**) mode II, and (**c**) mode III fracture tests.

Specimens were loaded using an Instron universal testing machine (5885) (Instron, Norwood, MA, USA) equipped with a 10 kN load cell. To load the specimens under pure mode I, a three-point bending setup with a span of 80 mm was used according to ASTM D5045 [42]. On the other hand, to load the specimens under pure mode II and mode III, the asymmetric four-point bending setup was utilized as discussed in detail in Refs. [41,44,45]. All the fracture tests were conducted under displacement control with a crosshead speed of 2 mm/min. Figure 3 shows the location of the supports and the experimental setups for the mode I, II, and III fracture tests.

It is worth mentioning that when performing fracture analyses using AE, it is important that geometry and setup parameters remain as constant as possible in order to examine the influence of loading mode variations without the effect of external factors. In fact, by selecting the pre-cracked prism-like specimen loaded using three-point and four-point bending, the effect of external parameters on the AE features is considered to be minimized, offering reliable analyses.

### 2.3. Acoustic Emission Technique

Acoustic emission is known as the propagation of elastic waves produced by releasing energy from a source within a material. When the wave reaches the surface of the specimen, it can be measured by a suitable sensor which is attached to the specimen’s surface. The mechanical disturbance can be transformed to a voltage using AE piezoelectric transducers. Since the mechanical disturbances due to elastic wave propagation are characterized by low energy, pre-amplification is needed [46]. AE is a passive non-destructive technique and is capable of detecting damage at early stages, prior to the final failure of the structure. Monitoring the damage evolution during the whole loading process of structures, without the need of having access to the whole surface or to all sides of the examined part, is a significant advantage of AE. Among the drawbacks of AE is the relatively low reproducibility of the test results, since the random nature of the fracture process is eventually reflected on the acquired AE waveforms. In addition, the AE waveform shape may be altered during propagation through the material (e.g., heterogeneity, reflections, attenuation) [47].

In the current study, AE activities were recorded using the AEWin (Physical Acoustic Corporation, West Windsor Township, NJ, USA, https://www.physicalacoustics.com/by-product/aewin/) software and the data acquisition Micro-II Digital AE system (Physical Acoustic Corporation, NJ, USA). PICO sensors, provided by Physical Acoustic Corporation (PAC) (Physical Acoustic Corporation, NJ, USA), were utilized. PICO sensors are broadband, resonant-type, single-crystal piezoelectric transducers with a wide operating frequency range, mostly between 200 and 800 kHz. Vaseline was applied to the surface of the sensor to facilitate acoustic coupling between the sensor and the samples. A threshold value of 35 dB was considered for all fracture tests in order to eliminate noise from the surroundings. In addition, the conventional pencil lead break test was conducted before each experiment to ensure the sensors were properly attached to the specimen and to calculate the material’s wave velocity. A 2/4/6 pre-amplifier (Physical Acoustic Corporation, NJ, USA) was used to amplify AE hits detected by sensors. The gain selector was set to 40 dB on the pre-amplifier. During the test, the AE signals were monitored by two sensors placed on the specimen as shown in Figure 3. In all cases, the sensors were positioned at the specimen’s middle width. Due to the inner supports for the mode III tests, the two sensors were placed at the same distance from the crack plane but on the opposite side of the specimen. Similar sensor configurations were thus considered for all three loading conditions to facilitate the comparison between AE activities.

**Figure 4:**
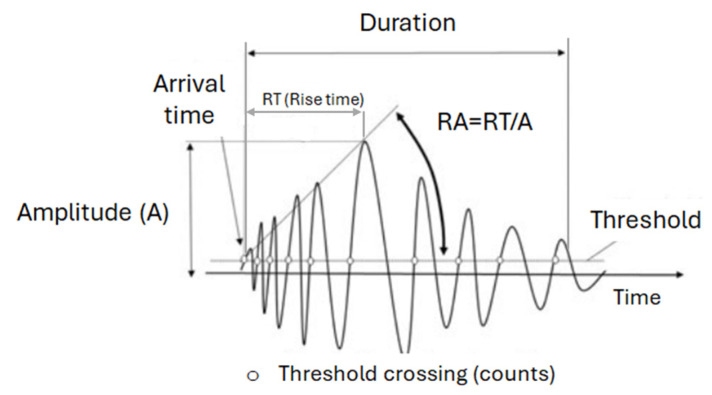
Typical AE burst signal and AE parameters [47].

After the experiments, all AE parameters were analyzed using the Noesis 5.7 (Physical Acoustic Corporation, NJ, USA) software. Among the AE parameters, the following ones were chosen for further examination: (1) the rise time (RT), expressed in μs, defined as the time from the first threshold crossing of a signal until its maximum amplitude, (2) the duration (DUR), expressed in μs, defined as the time difference between the first and last threshold crossings, (3) the average frequency (AF), expressed in kHz, defined as the number of total counts over the duration, (4) the peak frequency (PF), expressed in kHz, obtained from the Fourier transform of the waveform. It has to be noted that these parameters were chosen after analysis of the AE test results, first because both time and frequency domains are reflected by using them, and secondly due to their higher evident sensitivity to the different fracture modes compared to other features. Figure 4 depicts a typical AE burst signal and some of its different parameters, examined in this work.

In order to predict the fracture mode based on the AE signals, all the activity occurring from the beginning of the test until 90% of the maximum load was grouped and analyzed, with the reason behind this discussed in Section 3. AE hits are defined as the AE bursts detected by each sensor individually during the test. Apart from hits, AE events were also defined through linear localization between the two sensors. By knowing the wave velocity and the distance between the sensors, the AE events are defined as the AE hits that are captured from both sensors within a certain time frame and correspond to a specific damage incident. The results of the AE parameters analysis are discussed in Section 3.

### 2.4. Numerical Simulations

Several 2D elastic wave propagation simulations were conducted using the commercially available software Wave2000 (https://www.cyberlogic.org/wave2000.html) from Cyberlogic (CyberLogic Inc., New York, NY, USA) [48] to indicatively investigate the elastic wave propagation under mode I and mode II, and compare it with the experimental results. In order to simulate the wave propagation, the software solves Equation (1) using finite differences.

(1) $$\rho \frac{\partial w^{2}}{\partial^{2}t}=\left[\mu +\eta \frac{\partial }{\partial t}\right]\nabla^{2}w+\left[\lambda +\mu +\phi \frac{\partial }{\partial t}+\frac{\eta }{3}\frac{\partial }{\partial t}\;\right]\nabla \left(\nabla \circ w\right)$$

in which ρ, λ, μ, η, ϕ correspond to the density, first Lamé constant, second Lamé constant, shear viscosity, and bulk viscosity, respectively. Furthermore, ∇,∇∘ and ∂ are gradient, divergence, and partial differential operators, respectively. *T* and *w* denote time and displacement, respectively. In the simulations, the damping properties of the adhesive, namely the shear viscosity and bulk viscosity, were assumed to be 0.5 and 0.01 Pa·s, respectively. These numbers were derived from the Wave 2000 polymer materials library indicatively. All other values were experimentally obtained. Additionally, the air properties available in the Wave 2000 materials library were taken into consideration for the definition of the crack. The properties of air and the adhesive are listed in Table 2 and Table 3, respectively. Moreover, it has to be noted that no plasticity is considered for the adhesive. The results of the 2D wave propagation simulations for the mode I and mode II tests are provided in Section 4.

**Figure 5:**
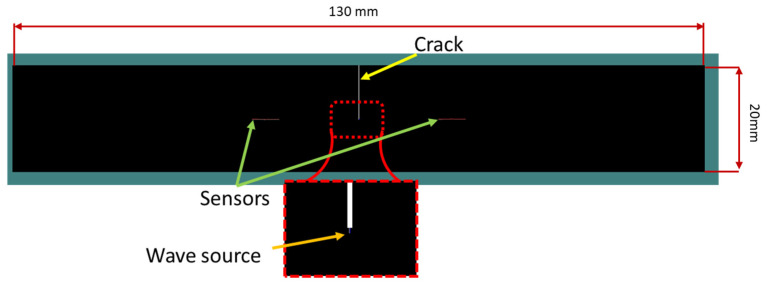
Geometry, crack, wave source, and sensors of the numerical model in the Wave 2000 software.

For conducting the simulations, the same geometry as the experimental one was considered. Figure 5 shows the geometry and sensors’ location in Wave 2000. It has to be noted that in Wave 2000 the sensors were modelled as simple lines due to software limitations. In reality, the applied PICO sensors have a circular shape with a 5 mm diameter.

**Figure 6:**
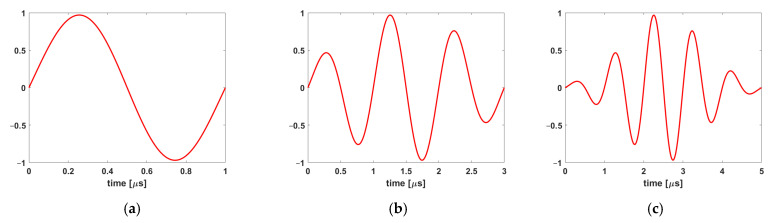
Gaussian sine pulse waveforms: (**a**) Case A, one cycle, (**b**) Case B, three cycles, and (**c**) Case C, five cycles.

The wave sources for mode I and mode II were defined using longitudinal and shear Gaussian sine pulses, respectively. The wave source was defined at the crack tip, and its length (along the crack plane, as shown in Figure 5) was equal to 0.1 mm. It has to be noted that 0.1 mm is chosen as an indication of the wave source at the crack tip. Table 4 lists the three distinct sine pulses that were considered for both the longitudinal and the shear sources during the numerical simulations. The short pulse (1 cycle, 1 μs duration, Case A) was initially used to produce clear results and provide separation of wave modes and reflections, while the longer pulses (Cases B and C) were applied to give a possibly more realistic representation of the experimentally received waveforms. Each applied Gaussian pulse waveform is indicatively illustrated in Figure 6.

## 3. Results

This section provides an overview of the results obtained from the fracture tests conducted under pure in-plane and out-of-plane loading conditions. First, the load-displacement curve of each fracture mode is represented. Since the objective of this study is to reveal the fracture mode prior to the final failure, the AE events from the start to 90% of the maximum load are extracted as a cluster and analyzed. Table 5 presents the load at the first event and the maximum load for two specimens per test performed under pure mode I, II, and III. It can be seen that the first event for mode I is recorded at less than 45% of the maximum load. On the other hand, for the mode II and mode III cases, the first event is usually recorded after 50% of the maximum load, indicating that the change of applied fracture mode exercises a certain influence on fracture behavior.

It has to be noted that since the acting load at the crack tip changes between the different modes, the maximum load value is also expected to be different. Particularly, during mode I loading, both bending moment and shear load are acting at the crack tip resulting in lower maximum load compared to the mode II and III conditions, during which solely shear load is acting at the crack tip.

**Figure 7:**
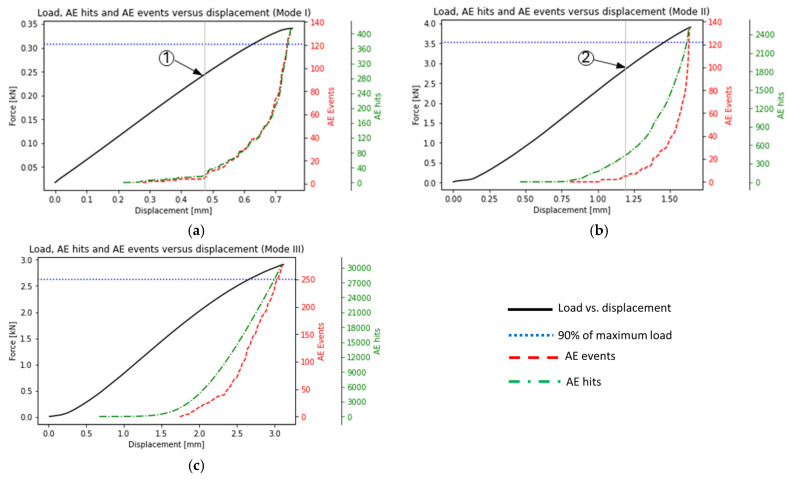
Load and number of AE hits and events versus displacement for (**a**) mode I, (**b**) mode II, (**c**) mode III tests.

Figure 7 illustrates the evolution of AE hits and events as well as the mechanical load versus displacement results for the three different cases, i.e., mode I, II, and III. For mode I, no significant amount of AE activities is observed between the first event registration and approximately 70% of the maximum load (see Figure 7a, point 1). Afterwards, the AE events increase significantly till the final failure of the specimen. In the mode II tests, almost no activities are recorded between the first event and until around 75% of the maximum load, whereas the AE events increase notably until the specimen fails (see Figure 7b, point 2). Contrary to the previous cases, during the mode III test, the AE activities follow an almost constant and ongoing increase after the first event and until the final failure (see Figure 7c). In all cases, the dotted horizontal line in Figure 7 represents 90% of the maximum load. The reasons for grouping and analyzing the events and hits until 90% of the maximum load are firstly to guarantee that the AE activities analyzed are registered quite before the final failure, and secondly, that the specimen is still intact and crack propagation has not been observed yet at this load level.

### 3.1. Event Localization Under In-Plane and Out-of-Plane Loading Conditions

**Figure 8:**
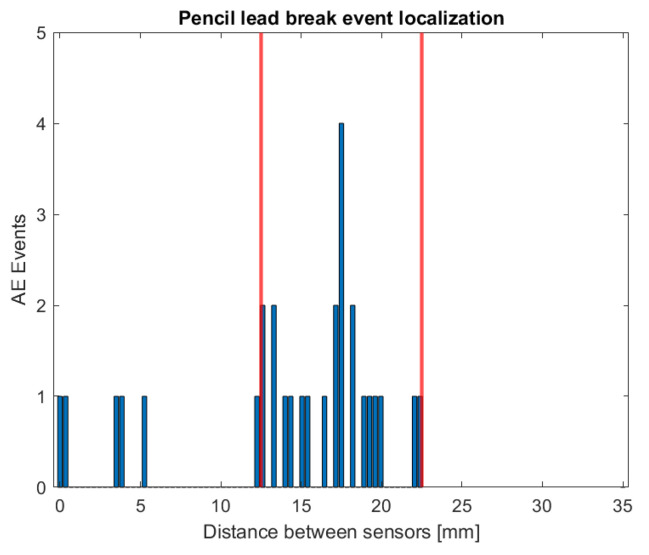
Manual AE source localization based on pencil lead breaks (distance between two sensors is 35 mm).

Utilizing multiple AE sensors allows one to perform localization and identify the wave source location by analyzing the time delay between signals acquired by different sensors. The pencil lead break test was employed to determine the appropriate distance margin for isolating the events. In order to achieve this, pencil lead breaks were conducted several times at the crack tip. Figure 8 depicts the results of the source localization of the pencil lead breaks at the crack tip. It has to be noted that even though the pencil lead breaks were performed with great care at the crack tip, and most of the events were localized reasonably close to the excitation point, i.e., at the middle distance between the two sensors, some events were localized several millimeters away from the expected excitation point. This scattered data is due to a number of factors, including specimen thickness, sensor size, and wave reflection.

**Figure 9:**
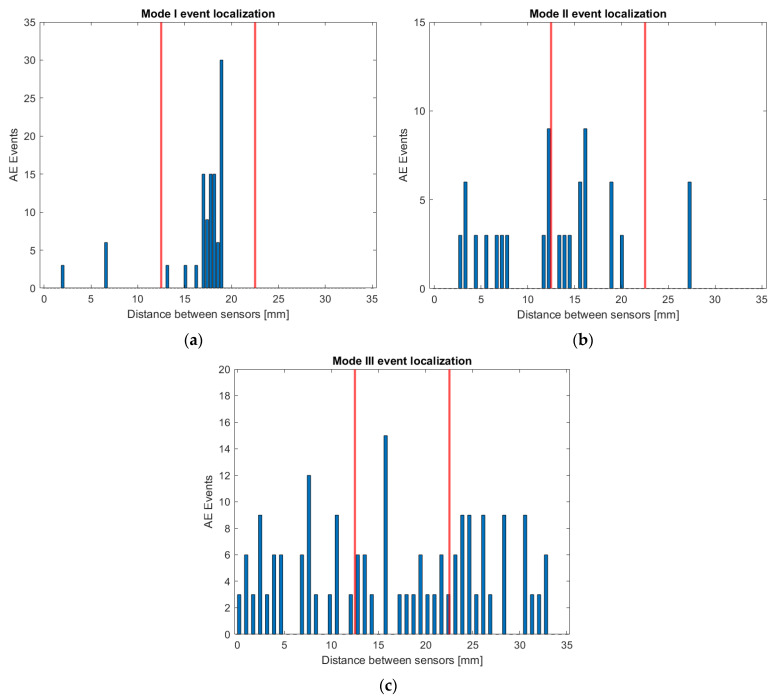
AE event localization for the (**a**) mode I, (**b**) mode II, and (**c**) mode III fracture tests.

Tsangouri and Aggelis examined the impact of sensor size on the properties of AE waves numerically [49]. They demonstrated that the size of the sensor can influence the amplitude of the signal, therefore one possible explanation for the scattered source localization in this study is the sensor size itself. Another potential reason is that the sensor size is not infinitesimal compared to the distance between the sensors (Pico sensor diameter is equal to 5 mm and its distance from the crack tip equals 17.5 mm). Additionally, AE waves are reflected upon reaching the boundaries, making accurate source localization challenging [50,51]. However, since the majority of the recorded events were localized within ±5 mm distance from the crack tip (see red vertical lines in Figure 8 and Figure 9), only events in this area were used for AE parameters analysis.

Moving to the mode I, II, and III fracture tests, AE events were obtained using linear (1D) localization between the two sensors, and the number of events versus the source distance is illustrated in Figure 9. The AE sources for mode I fracture are mainly concentrated at the middle distance between the two sensors, which is to be anticipated given that the pre-cracked section of the specimen is situated in the middle. In contrast, the scattering of the AE sources is greater during the mode II and III fracture tests. Apart from the above-mentioned factors that can generally affect the source localization, another potential reason for the higher scattering during the mode II and mode III tests is the friction of the mid-span loading rollers at the four-point bending setup (see Figure 3). This effect is attributed to the fact that the loading roller is positioned exactly at the top of the crack and further away from the sensors for the mode I test case, while the position of the rollers coincides with the position of the sensors regarding the mode II and III test setups.

### 3.2. AE Events Analysis Results

**Figure 10:**
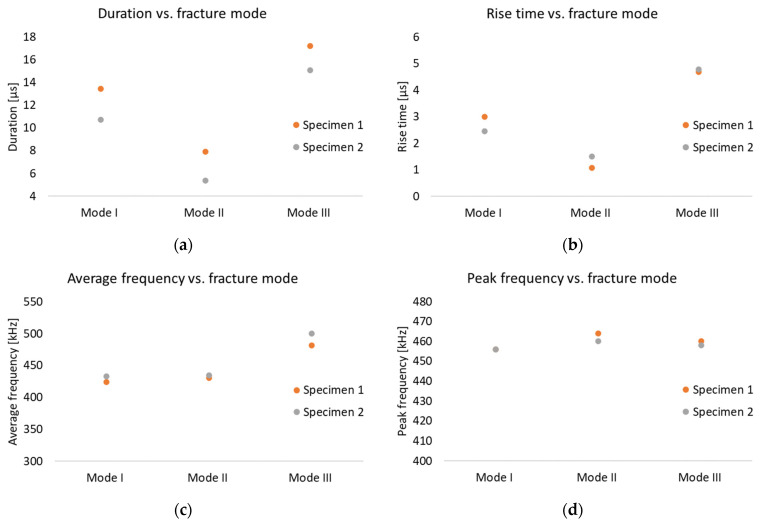
AE event parameters versus fracture mode: (**a**) duration, (**b**) rise time, (**c**) average frequency, and (**d**) peak frequency.

Figure 10 shows different AE parameters for different fracture modes as well as different specimens. It is clear that there are discernible variations in both the duration (Figure 10a) and rise time (Figure 10b) values when moving from mode I to mode II or mode III fracture, and that the fracture modes are distinguishable. On the contrary the examined frequency parameters, i.e., the average and peak frequency (Figure 10c and Figure 10d, respectively) cannot be clearly separated between the different fracture modes. Even if the average frequency shows an increase by changing the loading condition from in-plane to out-of-plane, the peak frequency does not significantly alter by changing the loading conditions of the cracked specimens.

Table 6 enumerates the AE parameters for each specimen tested under mode I, II, and III loading. Considering first the time parameters, by switching the loading conditions between the three different fracture modes, the rise time and duration values also change. The average duration values for mode I, II, and III are equal to 12.1, 6.6, and 16 μs, respectively. Regarding the rise time, the average values for mode I, II, and III are 2.73, 1.29, and 4.74 μs, respectively. This means that moving from mode I to mode II fracture results in a drop in both rise time and duration to their lowest values. Conversely, switching from mode II to mode III leads to an increase in both the rise time and duration. In summary, mode III fracture indicates the highest rise time and duration values, mode II shows the lowest ones, while mode I is in between. More importantly, there is no overlap between the AE time parameters for the different fracture modes and therefore the fracture mode is discernible in this study through AE analysis. Regarding the frequency parameters, the average frequency for modes I and II is equal to approximately 430 kHz, while it equals 491 kHz for mode III. This means that a difference between in-plane and out-of-plane fracture modes can be observed by comparing the average frequency values. In contrast, the peak frequency is approximately 450 kHz for all three different modes, offering no differentiation potential.

## 4. Discussion

To the best of the authors’ knowledge, there are no studies in the literature investigating the impact of fracture modes, including mode I, II, and III on the AE parameters in such detail that is done in this study revealing another capability of the AE in characterizing damage in polymers regarding of different loading conditions. This section discusses different aspects of the outcome of the current work.

### 4.1. AE Hits Analysis Results

In Section 3, the localized AE events were used. The events are generally considered the most reliable AE population of activity since they are localized and received by both sensors, practically eliminating noise effects [47]. Nevertheless, there might be exceptional circumstances in which it is impracticable to utilize two or more sensors or cases where the used software does not provide event localization capabilities. In this case, the AE hits should be used for interpretation of the AE results. Therefore, apart from the AE event analysis performed in this study, the AE hits recorded during the experiments were also grouped from the beginning of the test until 90% of the maximum load.

Table 7 shows the load at the first hit and the maximum load for each fracture mode. Compared to Table 5, which shows the load for the first event, the first hit is registered at a much earlier loading stage in all cases, especially for the mode II and mode III specimens. However, the trend of the recorded hits is similar to that of the events, shown in Figure 7.

**Figure 11:**
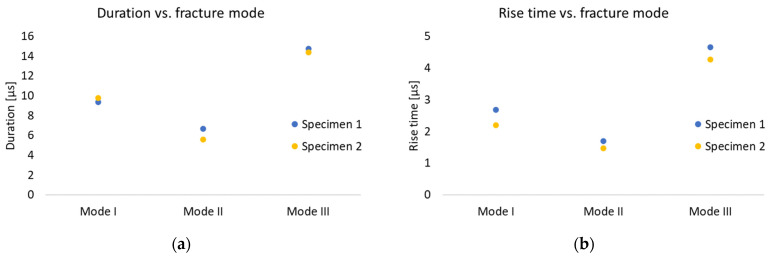
AE hit parameters versus fracture mode: (**a**) duration and (**b**) rise time.

Figure 11 depicts AE parameters extracted from the hits versus the fracture mode. A comparison between Figure 10 and Figure 11 reveals that the time parameters of AE hits exhibit a similar trend to the time parameters based on AE events, i.e., the fracture mode can be distinguished in both cases using time parameters, e.g., rise time and duration values. Also, by changing the loading conditions from mode I to mode II, both the rise time and the duration of the AE hits drop, while they increase when moving to mode III loading. Both for the events and for the hits analysis, the same trend is observed; mode III indicates the highest rise time and duration, mode II shows the lowest ones, and mode I is in between.

Table 8 presents the values of the AE parameters obtained by the hits analysis in all fracture tests. Similar values to those derived by the events analysis were obtained. The following conclusion can be drawn from the AE hits analysis: the average duration is equal to around 9.5, 6 and 14.5 μs, while the average rise time equals approximately 2.5, 1.5 and 4.5 μs, for mode I, II, and III fracture cases, respectively. Consequently, it can be concluded that both hits and events AE parameters can be used for fracture mode separation in fracture analysis of polymer materials.

### 4.2. Simulations Results

Shear-based AE activities are well-known to have a longer rise time compared to tensile-based events [25,47]. One reason in general is the larger percentage of energy emitted in the form of shear waves, which are generally slower than the longitudinal ones. Therefore, a considerable amount of energy arrives later in the shear waves, increasing the values of rise time and duration. This has been numerically simulated as well as experimentally supported in polymers and composite media among others [52,53]. However, in this study, the value of the rise time decreases when switching from mode I (tensile-based activities) to mode II (shear-based activities). This section presents the results of numerical simulations that consider shear and longitudinal wave sources, indicating that shear-based activities can lead to a shorter rise time.

**Figure 12:**
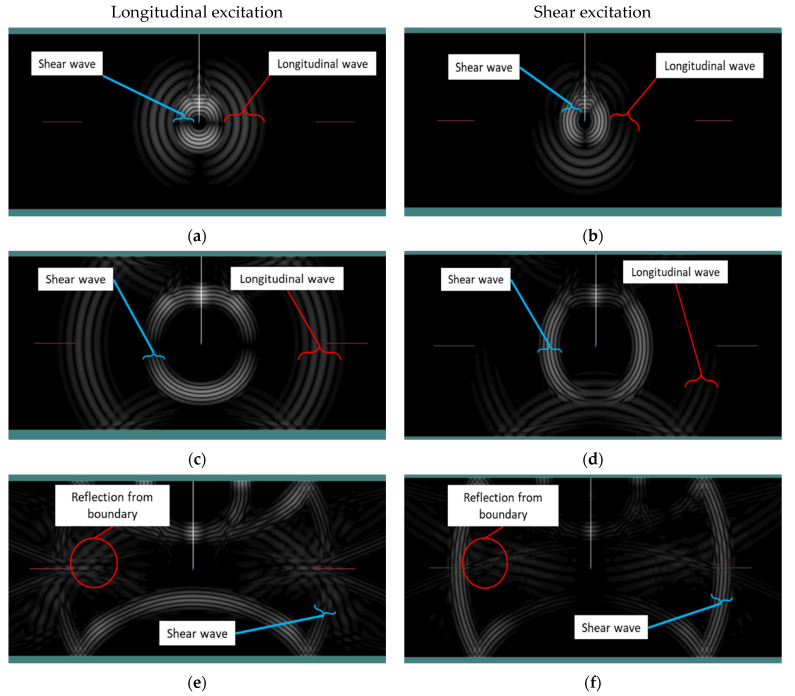
Wave propagation in the pre-cracked specimen for longitudinal and shear excitations: (**a**,**b**) development of the elastic wave after excitation, (**c**,**d**) arrival of the longitudinal wave to the sensors, and (**e**,**f**) arrival of the shear wave to the sensors.

Figure 12 illustrates the elastic wave propagation for the five-cycle excitation (see case C, Figure 6) for both longitudinal and shear excitations. As expected, first the longitudinal wave mode reaches the sensors. On the other hand, when the shear wave reaches the sensors, it intermingles with the reflection of the elastic wave from the boundaries. Furthermore, it can be seen that regardless of the excitation type, i.e., shear or longitudinal wave source, both shear and longitudinal waves are propagated through the material. For instance, in Figure 12a the wave source is longitudinal; however, both longitudinal and shear waves travel within the material. The same applies in Figure 12b, depicting the case of a shear wave excitation; both longitudinal and shear waves propagate through the medium. It is rational to conclude that regardless of wave source both longitudinal and shear waves will appear and propagate in the medium.

**Figure 13:**
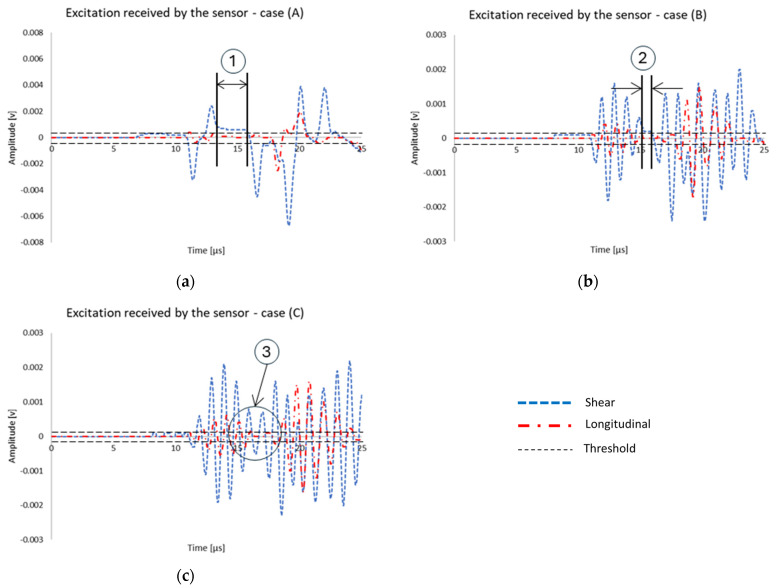
Simulated acoustic wave signals as received by the sensor for (**a**) one-cycle, (**b**) three-cycle, and (**c**) five-cycle excitation (threshold is shown by two horizontal dash lines).

Figure 13 illustrates the signal as received by the sensor from both the longitudinal and shear wave sources for all three distinct cases, i.e., one cycle, three cycles, and five cycles (see Figure 6). In all cases, it is clear that the amplitude of the received signal by the sensor is greater for the shear source compared to the longitudinal source. Table 9 presents the values of the rise time of the signal received by the sensor for both the shear and longitudinal wave sources for the three different excitation cases (see Figure 6). For case A or one cycle, the rise time of the shear wave is higher than the rise time of the longitudinal excitation. Conversely, in cases B and C, three and five cycles, respectively, the shear excitation has a shorter rise time compared to the longitudinal excitation. The threshold used for the determination of the rise time is shown with two dashed lines in Figure 13.

Polyzos et al. [54] investigated the effect of sensor distance from the source for shear and tensile cracks. It was demonstrated that when the sensor is close to the source, the different elastic waves, i.e., P-, S- and R- waves, are not separated. However, by increasing the distance between the sensor and the source, the different waves are well separated. In the present case, the short distance between the sensor and the source may be a potential contributing factor to the shorter rise time observed in mode II excitation compared to mode I because the different waves did not have sufficient time (or distance) to separate before reaching the sensors. This is also obvious from the results provided in Figure 13. For the one-cycle case, it can be seen that first the longitudinal wave arrives at the sensor and there is an interval between the arrival of the longitudinal and shear waves (see Figure 13, point 1). However, by increasing the number of cycles, this interval is reduced (see Figure 13, point 2). In the case of the five-cycle wave, it can be seen that the shear wave arrival coincides with the end of the longitudinal wave, pinpointing the mixing of different waves received by the sensor (see Figure 13, point 3). It has to be noted that the real AE signals acquired during the experiments are considerably longer than the numerical ones.

Section 3.2 demonstrates that AE events, being the reliable, localized sources, can be utilized to differentiate between various fracture modes in pre-cracked polymer materials. Section 4.1 presented a discussion and demonstrated the feasibility of using AE hits to identify fracture modes. Both events and hits analysis show that changing the fracture mode from I to III results in an increase in rise time. This result shows consistency with the existing studies in literature which present the same trend, i.e., increasing the rise time by switching from opening crack to shear crack [25]. The different waveform shapes and consequently the varying signal features are attributed to the micro-displacements taking place during fracture events that emit elastic waves of different modes. On the contrary, in the present study, by changing the loading conditions from mode I to mode II the reduction of the rise time is observed in both events and hits analysis of AE parameters. In order to understand this phenomenon, numerical simulations were performed and it was indicatively shown that for the same geometry and sensor location, the longitudinal excitation does not always lead to a shorter rise time. This discrepancy might be attributed to differences in the physical content of waveforms triggered by in-plane and out-of-plane shear fractures as well as to the small specimen geometry that does not allow sufficient propagation for the separation of different wave modes.

It is worth noting that the present study provided the first attempt for three-fracture mode recognition by AE parameters analysis. For example, neural networks can also be used for the clustering and training of the waveforms recorded from experiments to identify the fracture mode. Moreover, waveform-based analysis, such as wavelet transform, might be helpful to interpret the experimental results. Additionally, in the present research, 2D simulations of the elastic wave propagation were used; however, 3D simulations can provide more accurate understanding of the wave propagation under all three different fracture modes, which will be considered in future works. It has to be emphasized that this research does not account for parameters such as the impacts of plasticity at the crack tip or the existence of voids. Therefore, performing simulations taking into account these different parameters can also be considered in future works for developing improved models.

## 5. Conclusions

This study examines the capability of acoustic emission to identify all three different fracture modes in polymer materials from AE parameter analysis for the very first time. Fracture tests under mode I, mode II, and mode III were conducted using two AE sensors. It was observed that the AE parameters indicate changes in the loading conditions, proving the capability of AE for fracture mode characterization. The main findings regarding the impact of loading conditions on the AE parameters obtained from event analysis can be summarized as follows:

- The three different fracture modes, i.e., mode I, mode II, and mode III, could be effectively distinguished by AE parameters.
- Changing from in-plane (mode I and mode II) to out-of-plane (mode III) loading results in a considerable increase in the values of time-domain AE parameters such as rise time and duration.
- Smaller differences exist between mode I and mode II in time and frequency domain parameters.
- Numerical simulations were performed to understand wave mechanics and wave propagation as well as support experimental findings.

To the best of the authors’ knowledge, no studies have assessed the ability of acoustic emission to differentiate between fracture modes under both in-plane and out-of-plane loading conditions of polymer materials. Hence, it can be concluded that the most important outcome of the present research is the demonstration of AE’s capability to detect the fracture mode, apart from fracture surface inspection and optical methods like digital image correlation for recognizing the fracture mode. This AE ability can be combined with machine learning to investigate the fracture phenomena of massive structures like wind turbine blades. In order to do so, different research steps have to be undertaken, considering for instance scale effects, void inclusion, and the presence of mixed mode conditions. The current results can contribute to opening the way for the use of structural health monitoring in structures under combined loading, such as wind turbine blades.

## Figures and Tables

**Table 1:** Mechanical properties of the adhesive [43].

Sika Power^®^-830	
**Young’s modulus [GPa]**	2.8
**Yield strength [MPa]**	33
**Ultimate strength [MPa]**	40
**Poisson’s ratio**	0.4

**Table 2:** Properties of air used for the numerical simulations.

Air	
**Density [kg/m^3^]**	1.225
**First Lam*é* constant [Mpa]**	0.1
**Second Lam*é* constant [Mpa]**	0.01
**Shear viscosity [Pa·s]**	0.001
**Bulk viscosity [Pa·s]**	9.998 × 10^−8^

**Table 3:** Properties of the adhesive used for the numerical simulations.

Adhesive	
**Density [kg/m^3^]**	1150
**First Lam*é* constant [Mpa]**	4000
**Second Lam*é* constant [Mpa]**	1000
Shear viscosity * [Pa·s]	0.5
Bulk viscosity * [Pa·s]	0.01

* Values are taken from the Wave 2000 library from polymer materials.

**Table 4:** Details of the Gaussian sine pulses used as wave sources for numerical simulations.

	Case A	Case B	Case C
**Duration [μs]**	1	3	5
**Amplitude [−]**	1	1	1
**Frequency [MHz]**	1	1	1
**Number of cycles**	1	3	5

**Table 5:** First event and maximum load for specimens tested under mode I, II, and III.

	Mode I		Mode II		Mode III	
	S1	S2	S1	S2	S1	S2
**Maximum load [N]**	341	372	3904	3874	2908	2656
**First event load [N]**	151	155	1773	2212	1734	1358
**First event load as percentage of maximum load [%]**	44.3	41.6	45.4	57.1	59.6	51.1

**Table 6:** AE event parameters for mode I, II, and III loading.

	Mode I			Mode II			Mode III		
AE Parameter	Specimen		*Average*	Specimen		*Average*	Specimen		*Average*
	1	2		1	2		1	2	
**Duration [μs]**	10.7	13.5	*12.1*	5.4	7.9	*6.7*	15.1	17.2	*16.2*
**Rise time [μs]**	3.0	2.5	*2.8*	1.1	1.5	*1.3*	4.7	4.8	*4.8*
**Average frequency** **[kHz]**	424	433	*428*	431	435	*433*	482	500	*491*
**Peak frequency** **[kHz]**	456	456	*456*	464	460	*462*	460	458	*459*

**Table 7:** First hit and maximum load for specimens tested under mode I, II, and III.

	Mode I		Mode II		Mode III	
	S1	S2	S1	S2	S1	S2
**Maximum load [N]**	341	372	3904	3874	2908	2656
**First hit load [N]**	122	155	809	1409	456	63
**First hit load as percentage of maximum load [%]**	35.8	41.7	20.7	36.4	15.7	2.4

**Table 8:** AE hit parameters for mode I, II, and III loading.

	Mode I			Mode II			Mode III		
AE Parameter	Specimen		*Average*	Specimen		*Average*	Specimen		*Average*
	1	2		1	2		1	2	
**Duration [μs]**	9.3	9.8	*9.6*	6.6	5.6	*6.1*	14.8	14.4	*14.6*
**Rise time [μs]**	2.7	2.2	*2.5*	1.7	1.5	*1.6*	4.7	4.3	*4.5*

**Table 9:** Rise time of the signal emitted by longitudinal and shear sources as received by the sensor.

	Excitation Type	Case A	Case B	Case C
**Rise time (μs)**	Longitudinal (mode I)	7.312	8.233	8.809
	Shear (mode II)	8.194	6.525	7.427

## Data Availability

The data presented in this study are available on request from the corresponding author. The data are not publicly available due to privacy restrictions.

## References

[1] Bray D.E., Stanley R.K. (1997). Nondestructive Evaluation: A Tool in Design, Manufacturing, and Service.

[2] Ospitia N., Korda E., Kalteremidou K.A., Lefever G., Tsangouri E., Aggelis D.G. (2023). Recent Developments in Acoustic Emission for Better Performance of Structural Materials. Dev. Built Environ..

[3] Lima R.A.A., Tao R., Bernasconi A., Carboni M., Teixeira de Freitas S. (2024). Acoustic Emission Approach for Identifying Fracture Mechanisms in Composite Bonded Joints: A Study on Varying Substrate’s Stacking Sequence. Theor. Appl. Fract. Mech..

[4] Saeedifar M., Zarouchas D. (2020). Damage Characterization of Laminated Composites Using Acoustic Emission: A Review. Compos. B Eng..

[5] Hajikhani M., Ahmadi M., Farjpour M., Oskouei A.R., Sharifi A. (2011). Strain Energy Release Rate Assessment in Mode I Delamination of Foam Core Sandwich Composites by Acoustic Emission. J. Compos. Mater..

[6] Saeedifar M., Najafabadi M.A., Zarouchas D., Toudeshky H.H., Jalalvand M. (2018). Clustering of Interlaminar and Intralaminar Damages in Laminated Composites under Indentation Loading Using Acoustic Emission. Compos. B Eng..

[7] Zhao J., Guo Z., Lyu Q., Wang B. (2024). Prediction of Residual Compressive Strength after Impact Based on Acoustic Emission Characteristic Parameters. Polymers.

[8] Sobhani A., Saeedifar M., Najafabadi M.A., Fotouhi M., Zarouchas D. (2018). The Study of Buckling and Post-Buckling Behavior of Laminated Composites Consisting Multiple Delaminations Using Acoustic Emission. Thin-Walled Struct..

[9] Mohammadi R., Saeedifar M., Toudeshky H.H., Najafabadi M.A., Fotouhi M. (2015). Prediction of Delamination Growth in Carbon/Epoxy Composites Using a Novel Acoustic Emission-Based Approach. J. Reinf. Plast. Compos..

[10] Guo Y., Zhu S., Chen Y., Liu D., Li D. (2019). Acoustic Emission-Based Study to Characterize the Crack Initiation Point of Wood Fiber/HDPE Composites. Polymers.

[11] Heidary H., Ahmadi M., Rahimi A., Minak G. (2012). Wavelet-Based Acoustic Emission Characterization of Residual Strength of Drilled Composite Materials. J. Compos. Mater..

[12] Mohan R., Prathap G. (1980). An Acoustic Emission Energy Analysis and Its Use to Study Damage in Laminated Composites. J. Nondestruct. Eval..

[13] Fotouhi M., Sadeghi S., Jalalvand M., Ahmadi M. (2015). Analysis of the Damage Mechanisms in Mixed-Mode Delamination of Laminated Composites Using Acoustic Emission Data Clustering. J. Thermoplast. Compos. Mater..

[14] Boominathan R., Arumugam V., Santulli C., Adhithya Plato Sidharth A., Anand Sankar R., Sridhar B.T.N. (2014). Acoustic Emission Characterization of the Temperature Effect on Falling Weight Impact Damage in Carbon/Epoxy Laminates. Compos. B Eng..

[15] Kalteremidou K.A., Murray B.R., Carrella-Payan D., Cernescu A., Van Hemelrijck D., Pyl L. (2021). Failure Analysis of CF/Epoxy Hollow Beam Components Using Digital Image Correlation and Acoustic Emission Analyses. Compos. Struct..

[16] Kalteremidou K.A., Aggelis D.G., Van Hemelrijck D., Pyl L. (2021). On the Use of Acoustic Emission to Identify the Dominant Stress/Strain Component in Carbon/Epoxy Composite Materials. Mech. Res. Commun..

[17] Zarouchas D., Van Hemelrijck D. (2014). Mechanical Characterization and Damage Assessment of Thick Adhesives for Wind Turbine Blades Using Acoustic Emission and Digital Image Correlation Techniques. J. Adhes. Sci. Technol..

[18] Crawford A., Droubi M.G., Faisal N.H. (2018). Analysis of Acoustic Emission Propagation in Metal-to-Metal Adhesively Bonded Joints. J. Nondestruct. Eval..

[19] Galanopoulos G., Milanoski D., Eleftheroglou N., Broer A., Zarouchas D., Loutas T. (2023). Acoustic Emission-Based Remaining Useful Life Prognosis of Aeronautical Structures Subjected to Compressive Fatigue Loading. Eng. Struct..

[20] Crawford A.R., Droubi M.G., Faisal N.H. Modal acoustic emission analysis of mode-I and mode-II fracture of adhesively-bonded joints. Proceedings of the 33rd European Working Group on Acoustic Emission (EWGAE) European Acoustic Emission Testing Conference (EWGAE 2018).

[21] Dzenis Y.A., Saunders I. (2002). On the Possibility of Discrimination of Mixed Mode Fatigue Fracture Mechanisms in Adhesive Composite Joints by Advanced Acoustic Emission Analysis. Int. J. Fract..

[22] Saeedifar M., Saleh M.N., Krairi A., de Freitas S.T., Zarouchas D. (2023). Structural Integrity Assessment of a Full-Scale Adhesively-Bonded Bi-Material Joint for Maritime Applications. Thin-Walled Struct..

[23] Du J., Wang H., Cheng L., Bi Y., Yang D. (2023). Damage Localization, Identification and Evolution Studies during Quasi-Static Indentation of CFRP Composite Using Acoustic Emission. Polymers.

[24] Linn D.M., Aggelis D.G., Tsangouri E. (2022). Single-Lap Shear Tests of Textile Reinforced Mortar Retrofit Systems Bonded to Masonry: Revealing the Fracture Progress by Digital Image Correlation and Acoustic Emission. Mater. Struct..

[25] Ohno K., Ohtsu M. (2010). Crack Classification in Concrete Based on Acoustic Emission. Constr. Build. Mater..

[26] Liu X., Zeng Y., Xia C., Liu H., Xie Q., Zhong Y. (2023). Influence of Specimen Size on Granite Fracture Characteristics and Acoustic Emission Phenomena under Mode I Loading Conditions. Theor. Appl. Fract. Mech..

[27] Livitsanos G., Shetty N., Hündgen D., Verstrynge E., Wevers M., Van Hemelrijck D., Aggelis D.G. (2018). Acoustic Emission Characteristics of Fracture Modes in Masonry Materials. Constr. Build. Mater..

[28] Farhidzadeh A., Mpalaskas A.C., Matikas T.E., Farhidzadeh H., Aggelis D.G. (2014). Fracture Mode Identification in Cementitious Materials Using Supervised Pattern Recognition of Acoustic Emission Features. Constr. Build. Mater..

[29] Betteridge D., Connors P.A., Lilley T., Shoko N.R., Cudby M.E.A., Wood D.G.M. (1983). Analysis of Acoustic Emissions from Polymers. Polymer.

[30] Lainé E., Grandidier J.-C., Gorge A.-L., Cruz M., Maziers E. Identification of damages into a polymer structure by ae-transition from tensile specimen to structure. Proceedings of the EWGAE35 & ICAE10 Conference on Acoustic Emission Testing.

[31] Casiez N., Deschanel S., Monnier T., Lame O. (2014). Acoustic Emission from the Initiation of Plastic Deformation of Polyethylenes during Tensile Tests. Polymer.

[32] Heinzmann R., Seghir R., Alam S.Y., Réthoré J. (2024). Investigation of Fracture Source Mechanisms through Full-Field Imaging and Acoustic Emission. Eng. Fract. Mech..

[33] Skal’s’kii V.R., Makeev V.F., Stankevich O.M., Kyrmanov O.S., Vynnyts’ka S.I., Opanasovich V.K. (2015). Strength Evaluation of Stomatologic Polymers by Wavelet Transform of Acoustic Emission Signals. Strength Mater..

[34] Guo J., Doitrand A., Sarr C., Chataigner S., Gaillet L., Godin N. (2022). Numerical Voids Detection in Bonded Metal/Composite Assemblies Using Acousto-Ultrasonic Method. Appl. Sci..

[35] Hamam Z., Godin N., Fusco C., Doitrand A., Monnier T. (2021). Acoustic Emission Signal Due to Fiber Break and Fiber Matrix Debonding in Model Composite: A Computational Study. Appl. Sci..

[36] Yang H., Wang B., Grigg S., Zhu L., Liu D., Marks R., Yang H., Wang B., Grigg S., Zhu L. (2022). Acoustic Emission Source Location Using Finite Element Generated Delta-T Mapping. Sensors.

[37] Zeinedini A. (2019). A Novel Fixture for Mixed Mode I/II/III Fracture Testing of Brittle Materials. Fatigue Fract. Eng. Mater. Struct..

[38] Richard H.A., Schramm B., Schirmeisen N.H. (2014). Cracks on Mixed Mode Loading—Theories, Experiments, Simulations. Int. J. Fatigue.

[39] He M.Y., Hutchinson J.W. (2000). Asymmetric Four-Point Crack Specimen. J. Appl. Mech..

[40] Karami J., Ayatollahi M.R., Saboori B. (2019). Fracture Study of MWCNT/Epoxy Nanocomposite under Pure Mode III Loading Using Anti-Symmetric Four-Point Bend Specimen. Mater. Des. Process. Commun..

[41] Karami J., Ayatollahi M.R., Saboori B. (2020). Experimental Fracture Investigation of CNT/Epoxy Nanocomposite under Mixed Mode II/III Loading Conditions. Fatigue Fract. Eng. Mater. Struct..

[42] (2014). Standard Test Methods for Plane-Strain Fracture Toughness and Strain Energy Release Rate of Plastic Materials.

[43] Kojouri A.S., Karami J., Kalteremidou K.-A., Fan J., Sharma A., Vassilopoulos A.P., Michaud V., Van Paepegem W., Van Hemelrijck D. (2024). An Experimental and Analytical Study of Mode I Fracture and Crack Kinking in Thick Adhesive Joints. Compos. B Eng..

[44] Karami J., Kojouri A.S., Fan J., Kalteremidou K.-A., Wim Paepegem V., Vassilopoulos A., Michaud V., Van Hemelrijck D. Investigating the Effects of Different Crack Sharpening Methods on the Mode I and Mode II Fracture Tests of an Epoxy Resin. Proceedings of the 20th European Conference on Composite Materials.

[45] Kojouri A.S., Karami J., Fan J., Kalteremidou K.-A., Vassilopoulos A., Van Paepegem W., Michaud V., Van Hemelrijck D. Fracture of Structural Adhesive Under Pure Mode III Loading Conditions: Experimental Study and Challenges. Proceedings of the 20th European Conference on Composite Materials.

[46] Nguyen A.D., Godinez V. (2009). Integrated Health and Corrosion Monitoring Systems in the Aerospace Industry. Corrosion Control in the Aerospace Industry.

[47] Grosse C.U., Ohtsu M., Aggelis D.G., Shiotani T. (2022). Acoustic Emission Testing.

[48] CyberLogic Wave2000 Software. https://www.cyberlogic.org.

[49] Tsangouri E., Aggelis D.G. (2018). The Influence of Sensor Size on Acoustic Emission Waveforms—A Numerical Study. Appl. Sci..

[50] Romhány G., Czigány T., Karger-Kocsis J. (2017). Failure Assessment and Evaluation of Damage Development and Crack Growth in Polymer Composites Via Localization of Acoustic Emission Events: A Review. Polym. Rev..

[51] Kundu T. (2014). Acoustic Source Localization. Ultrasonics.

[52] Gresil M., Saleh M.N., Soutis C. (2016). Transverse Crack Detection in 3D Angle Interlock Glass Fibre Composites Using Acoustic Emission. Materials.

[53] Sause M., Hamstad M. (2018). 7.14 Acoustic Emission Analysis. Comprehensive Composite Materials II.

[54] Polyzos D., Papacharalampopoulos A., Shiotani T., Aggelis D.G. (2011). Dependence of AE Parameters on the Propagation Distance. J. Acoust. Emiss..

